# Chronic Insulin Exposure Induces ER Stress and Lipid Body Accumulation in Mast Cells at the Expense of Their Secretory Degranulation Response

**DOI:** 10.1371/journal.pone.0130198

**Published:** 2015-08-11

**Authors:** William E. Greineisen, Kristina Maaetoft-Udsen, Mark Speck, Januaria Balajadia, Lori M. N. Shimoda, Carl Sung, Helen Turner

**Affiliations:** 1 Laboratory of Immunology and Signal Transduction, Chaminade University, Honolulu, Hawaii, United States of America; 2 Department of Cell and Molecular Biology, John A. Burns School of Medicine, University of Hawaii, Honolulu, Hawaii, United States of America; Fundação Oswaldo Cruz, BRAZIL

## Abstract

Lipid bodies (LB) are reservoirs of precursors to inflammatory lipid mediators in immunocytes, including mast cells. LB numbers are dynamic, increasing dramatically under conditions of immunological challenge. We have previously shown *in vitro* that insulin-influenced lipogenic pathways induce LB biogenesis in mast cells, with their numbers attaining steatosis-like levels. Here, we demonstrate that *in vivo* hyperinsulinemia resulting from high fat diet is associated with LB accumulation in murine mast cells and basophils. We characterize the lipidome of purified insulin-induced LB, and the shifts in the whole cell lipid landscape in LB that are associated with their accumulation, in both model (RBL2H3) and primary mast cells. Lipidomic analysis suggests a gain of function associated with LB accumulation, in terms of elevated levels of eicosanoid precursors that translate to enhanced antigen-induced LTC4 release. Loss-of-function in terms of a suppressed degranulation response was also associated with LB accumulation, as were ER reprogramming and ER stress, analogous to observations in the obese hepatocyte and adipocyte. Taken together, these data suggest that chronic insulin elevation drives mast cell LB enrichment *in vitro* and *in vivo*, with associated effects on the cellular lipidome, ER status and pro-inflammatory responses.

## Introduction

In adipocytes and hepatocytes, lipid droplets (LD) are structures specialized for lipid storage in support of energy production [1–4]. Related structures, the lipid bodies (LB), are found in leukocytes and are additionally specialized as reservoirs of bioactive lipid precursors [5, 6]. In mast cells, and other immune system cells, LB are sites for arachidonic acid metabolism, eicosanoid synthesis and storage [2, 6–14]. Thus the potential for a mast cell to generate pro-inflammatory mediators such as leukotriene C4 (LTC4) may be related to the size of the pool of precursors contained in these LB [5].

LB numbers are regulated in macrophages and other granulocytes in response to bacterial and parasitic infection [15, 16] and other inflammatory states (e.g. arthritic joint leukocytes [17], lavage from patients with acute respiratory distress [18], sepsis [19, 20] and mycobacterial infection [21]). Expansion of the LB pool in neutrophils and eosinophils has been correlated with enhancement in pro-inflammatory lipid release, but LB in macrophages can also simply represent accumulation of absorbed dietary lipids. Chronic insulin elevation is a third scenario that causes LB expansion *in vitro* in a leukocyte, the mast cell [22]. However, further studies are required to establish whether a similar phenotype is engendered by a positive energy balance and hyperinsulinemia *in vivo*. This *in vitro* lipogenesis has been associated with enhanced *de novo* synthesis of mediators such as LTC4 in response to antigenic stimulation [22]. However, in the absence of any published lipidomic analysis of these LB, we cannot yet state whether these structures are primarily reservoirs of absorbed dietary lipid (c.f. foam cells) or of *de novo* synthesized bioactive lipid precursors induced by innate stimuli in granulocytes.

The impact of a LB-rich phenotype on mast cell function may extend beyond alterations in cellular lipid content. In adipocytes and hepatocytes, steatosis is an adapted state that alters cell status [23]. For example, cellular steatosis in the obese liver is associated with induction of ER stress, and reprogramming of the ER towards lipid rather than protein synthesis [24–27]. ER distension and dysregulation of the ER calcium store have also been noted [28, 29]. All of these adaptations are likely to affect cellular responses to incoming signals, as is the highly oxidative cytoplasmic environment documented in LB-rich cells [30]. Steatosis in foam cells is associated with altered cytokine profiles, phagocytic capacity and signalling responses to bacterial ligands [6, 31]. The consequences of mast cell steatosis for functional responses to antigen require assessment, particularly in light of our previous *in vitro* data suggesting that degranulation of histamine-bearing granules may be suppressed in LB-enriched mast cells [22].

Here, we characterized the LB population that accumulates in mast cells chronically exposed to insulin. Enrichment for LB was observed in the model mast cell line RBL2H3, peripheral blood basophils and in primary bone marrow derived mast cells (BMMC) under *ex vivo* or *in vivo* exposure to high fat diet (HFD)-induced hyperinsulinemia. HFD/hyperinsulinemic conditions *in vivo* are associated with gains and losses of function in mast cells/basophils (elevated LTC4 release and suppressed secretory granule degranulation). We describe the first lipidome for LB isolated from mast cells, and offer the new direct evidence that these LB are enriched in precursor pools for bioactive lipid mediators. The accumulation of large numbers of cytosolic LB is sufficient to shift the whole cell lipidome to a nominally more ‘pro-inflammatory’ state. This lipidomic fingerprint also provides evidence for both overlapping and discrete storage functions of immunocyte LB when compared to the lipid content of adipocyte lipid droplets. Finally, LB accumulation in response to chronic insulin elevation induces ER lipid accumulation and ER stress in mast cells, analogously to alterations seen in the obese hepatocyte and adipocyte. Taken together, these data suggest that chronic insulin exposure drives a steatosis-like LB accumulation in mast cells, with marked and selective effects on their pro-inflammatory outputs.

## Materials and Methods

### Cell culture

RBL2H3 from ATCC (CRL-2256) were grown at 37°C, 5% CO_2_, in 95% humidity in Dulbecco’s Modification of Eagle Medium (Mediatech Inc., Herndon, VA) with 10% heat-inactivated Fetal Bovine Serum (Mediatech) and 2mM Glutamine. Murine bone marrow derived mast cells (BMMC) were generated by culturing femoral bone marrow cells from C57 BL6 mice in RPMI supplemented with 10% FBS, 2mM l-Gln, 2mM NEAA, 1mM Sodium pyruvate, 50 micromolar 2-mercaptoethanol, and 5ng/ml IL-3 at 37°C, 5% CO_2_, 95% humidity for 5–6 weeks. Peripheral blood basophils were purified by MACS (Miltenyi Biotech) and maintained briefly in RPMI as described above.

### Chemicals

General chemicals were from VWR (West Chester, PA). Phorbol 12,13 myristate acetate (PMA) and ionomycin were from Calbiochem (Gibbstown, NJ). Antibodies to the following epitopes were sourced as follows: ATF6 (Abcam, Cambridge, MA); phospho PERK-Thr380, IRE1 alpha, Calnexin, perilipins A and B, ATG7, ATG12, LC3A, LC3B and Grb2 (Cell Signalling, Danvers, MA). Oil Red O and KLH-DNP were from EMD (Gibbstown, NJ). IgE anti-DNP were from Sigma. 4′,6-Diamidino-2-Phenylindole (DAPI), Alexa- and HRP conjugated secondary antibodies were from Invitrogen (Temecula, CA) and Amersham (Piscataway, NJ). Insulin was added at 0.01 mg/ml where indicated in the presence of 10% FBS, 0.25 micromolar Dexamethasone, 0.5 micromolar IBMX (FDI).

### High fat diet fed mice

Eight-week old C57 BL6 male mice were fed *ad libitum* on high fat diet (60% fat calories, BioServ S3282) for 8 weeks (Dr. A Stokes, University of Hawaii). Littermate controls were fed normal chow (12% fat calories, LabDiet 5001). HFD-induced hyperinsulinemia was confirmed by measurement of plasma insulin (Linco St Charles, MO), in control and HFD-treated animals. Femurs were collected from euthanized mice by CO_2_ asphyxiation and preparation of bone marrow derived mast cells was performed (see cell culture). All procedures were approved by the Institutional Animal Care and Use Committee of the University of Hawaii.

### ORO Staining

Cells (50,000 per cm^2^) were fixed on coverslips (0.4% (w/v) paraformaldehyde 1h, RT), washed twice with tap water and stained with Oil Red O (ORO, 0.35% in 6:4 EtOH:water, 15 min RT followed by two dH_2_O washes). Coverslips were mounted in Crystal-Mount (Electron Microscopy Sciences, Hatfield, PA). Live cell experiments comprised 30 min staining at 37°C in media with 4 micromolar Fluo-4 and 0.05% ORO from 5% stock in 70:30 water:ethanol.

### Imaging

Bright field and fluorescence imaging of cells in MatTek dishes (50,000 cells per cm^2)^ were performed on a Nikon Ti Eclipse C1 epi-fluorescence and confocal microscopy system, equipped with heated stage. Available laser lines in FITC, TxRed and Cy5 were supplied by a 488nm 10mW solid state laser, a 561nm 10mW diode pump solid state (DPSS) laser and a 638nm 10mW modulated diode laser. Z stack size was 15 microns. Each z disc (optical section) was 150 nm. Pinhole size for all images was 60 microns. Images were analysed in NIS Elements (Nikon, Melville, NY).

### Electron Microscopy (EM)

Cell pellets were resuspended 1:1 in 4% formaldehyde/1% glutaraldehyde for two rounds of fixation. Fixatives were replaced with 8% (0.2M) sucrose in 0.1 M PBS (3x15 min) and cells were post-fixed with 1% OsO_4_ in PBS (1 h) then rinsed in 0.1 M PBS. Dehydrations for 15 min each in 0, 70, 95 and 100% EtOH were performed, followed by 100% Propylene oxide (2 x 15min), 1:1 EMBed 812 and Propylene Oxide (1 h) and 2:1 EMBed 812:Propylene Oxide (16h in dessicator). Embedding was performed with Embed in Beam capsules (60°C/48 h). Ultrathin sections were produced and grids were treated with uranyl acetate (15 min, dH_2_O rinse) and lead citrate (5 min, dH_2_O rinse). EM images were produced by IHC World (Woodstock, MD) and the Biological EM Facility at the University of Hawaii.

### Cell Stimulations

Fc-epsilonRI stimulation used 100 ng/ml IgE anti-DNP (16 h/ 37°C) followed by three washes and the addition of 250 ng/ml KLH-DNP for indicated times. PMA and ionomycin were both used at 500nM. Insulin was used in isolation at 0.2–10 μg /ml or in combination with dexamethasone and IBMX at 0.05μM and 0.025 μM in the presence of 15% FBS (Insulin-FDI). BMMC from HFD mice were additionally *in vitro* stimulated with a sub-optimal dose of insulin (insufficient to induce LB biogenesis by itself in control experiments) of 200 ng/ml.

### Cell Lysis and Western blots

Cells were lysed (ice/30 min) in 350μl of lysis buffer (50mM Hepes pH 7.4, 250mM NaCl, 20mM NaF, 10mM iodoacetamide, 0.5%(w/v) Triton X100, 1mM PMSF (phenylmethylsulfonylfluoride), 500 mg/ml aprotinin, 1.0 mg/ml leupeptin and 2.0 mg/ml chymostatin). Lysates were clarified (17,000*g*, 20 min) and acetone precipitated (1.4 volumes acetone, 1h/–20°C, followed by 10,000g/5 min). Protein determination used a *Dc* protein determination kit (BioRad, Temecula, CA) with BSA as a standard. Protein was resolved by 10% reducing SDS-PAGE in a modified Laemmli buffer and electro-transferred to PVDF in 192mM glycine, 25mM Tris (pH 8.8). Membranes were blocked (5% non-fat milk in PBS, 1h, RT) and probed (primary antibodies in PBS/0.05% Tween-20/0.05% NaN_3_, 16 h/4°C). Developing antibodies comprised anti-rabbit or anti-mouse IgGs conjugated to horseradish peroxidase (Amersham) at 0.1 micrograms/ml in PBS/0.05% Tween-20 (45 min/RT). Signal was visualized using ECL (Amersham) and Kodak BioMax film. Films were scanned at >600 dpi and quantification was performed using Image J (NIH).

### ER/microsomal and lipid body preparation by ultracentrifugation

ER/microsomal fractions were purified using an ER Isolation kit (Sigma). Enrichment was assessed by anti-Calnexin Western blot. ER protein and lipid levels were assayed by BCA analysis, and Bligh-Dyer extraction followed by ORO analysis using absorbance spectroscopy, respectively [32].

### Staining for lipid content

Cells were fixed with 0.4% (w/v) paraformaldehyde (1h, RT), washed twice with dH2O and stained with Oil Red O (ORO, 0.35% in 6:4 EtOH:water, 15 min RT) followed by two dH2O washes or other dyes as indicated. Coverslips were mounted in Crystal-Mount (Electron Microscopy Sciences, Hatfield, PA). Imaging was performed on a Nikon Ti Eclipse, acquired through a Plan Apo VC 100X 1.40 oil objective, and analysed in NIS Elements.

### Beta Hexosaminidase assay for degranulation

Cells were plated in 12-well cluster plates at 10^5^ cells/well in 230 microliters/Tyrode’s buffer. After 30 min at 37°C, 25 μsupernatant was removed, clarified by micro-centrifugation, and transferred to a 96-well plate containing 100 μl (per well) *p*-NAG (1 mM *p*-*N*-acetyl glucosamine (Sigma) in 0.05 M citrate buffer (pH 4.5) substrate solution. After 1.5 hr at 37°C, reactions were quenched by addition of 100 microliters of 0.2 M glycine (pH 9.0). Beta hexosaminidase levels were read as A405 nm in a Benchmark plate reader (BioRad, Hercules, CA). Results are reported as the mean (± SD) of triplicate samples.

### mRNA analysis

RNA was extracted from RBL2H3 (Qiagen, Hilden, Germany). Agilent RNA Spike-In, One Color Mix (Agilent Technologies, Santa Clara, USA) was prepared and 100ng RNA was converted into cRNA and labeled with cyanine-3 with Low Input Quick Amp Labelling Kit (Agilent). Samples were purified using RNeasy mini columns (Qiagen) and cRNA was assessed using Nanodrop (ND-2000C). Using the one-color protocol, equal amounts of Cy3-labeled cRNA (1500 ng) from stimulated and non-treated cells were hybridized in duplicates to Agilent Whole Rat Genome Microarray 4x44K (G4131F) for 17 hrs at 65°C. The microarray comprised of 45,000 probes, representing >41,000 transcripts. Hybridized microarrays were washed, scanned and analyzed (Agilent G2565CA, Agilent Genespring v11.5). Intensities were scaled to the median of all samples and no baseline normalization was performed. Fold changes with associated p-values were calculated. For statistical analyses, an unpaired t-test was performed, using Benjamini-Hochberg for multiple testing amongst the duplicates.

### Leukotriene C4 Assay

Cells were treated as indicated and stimulated via the FcεRI or using PMA/ionomycin. After 1h, supernatants were assayed for LTC4 using an EIA kit (Cayman Chemicals, Ann Arbor, MI) in reference to a standard curve. Color development proceeded for 45 min and absorbance was read as A405 nm. Results are reported as the mean (± SD) of triplicates.

### ER/microsomal and lipid body preparation by ultracentrifugation

ER/microsomal fractions were purified using an ER Isolation kit (Sigma). Enrichment was assessed by anti-Calnexin Western blot. ER protein and lipid levels were assayed by BCA analysis, and Bligh-Dyer extraction followed by ORO analysis using absorbance spectroscopy, respectively [32].

### Lipid body isolation

Cells were resuspended in disruption buffer (DB, 25 mM Tris-HCl, 100 mM KCl, 1 mM EDTA, 5 mM EGTA, pH of 7.4) and Dounce homogenized. The cavitate was mixed dropwise 1:1 with DB containing 1.08 mol/L sucrose, and centrifuged (1500*g*/10 min). Supernatant was sequentially overlaid with 0.27mol/L and 0.135mol/L sucrose buffers and TOP solution (25mM Tris, 1mM EDTA, 1mM EGTA, pH 7.4), and then centrifuged at 150,500*g* for 60 min at 4°C. Fractions of 575 microliters were collected from top to bottom: buoyant lipid bodies, mid-zone, microsomal pellet and nuclei. Fractions were sonicated in TOP and protein was measured by BCA assay. Western blot confirmation of perilipin enrichment in LB fractions and sham purifications were performed using ORO to track LB in the density gradient.

### Lipidomic analysis

Lipids were extracted in the presence of authentic internal standards by the Folch method using chloroform:methanol (2:1 v/v) [33]. For separation of neutral lipid classes (free fatty acids, triacylglycerol, diacylglycerol, free cholesterol and cholesterol esters), a solvent system consisting of petroleum ether/diethyl ether/acetic acid (80:20:1) was employed. Individual phospholipid classes were separated by liquid chromatography (Agilent Technologies). Whole cell and microaspirated lipid body lipidomic analysis was performed in collaboration with Metabolon Inc. Heatmaps were generated using the RColorBrewer and gplots packages in R [32, 34]. Relative abundance (nmol%) was calculated from the amount of each fatty acid among individual lipid groups detected in the LBs. Ribbon plots of the LB relative abundance data were visualized using Circos [17]. For ER lipid analysis each lipid class was transesterified in 1% sulfuric acid in methanol in a sealed vial under a nitrogen atmosphere at 100°C for 45 min. Fatty acid methyl esters (FAME) were extracted with hexane/0.05% butylated hydroxytoluene and sealed under nitrogen. FAME were separated and quantified by capillary GC (Agilent Technologies model 6890) equipped with a 30 m DB 88 capillary column and a flame ionization detector. ER lipid analysis was performed with Dr. Shari Forbes (University of Ottowa Institute of Technology).

### Analysis

Results are shown as the mean ± standard deviation. Statistical significance was determined based on ANOVA or Student's t-test where appropriate. Adjacent to data points in the respective graphs, significant differences were recorded as follows: single asterisk, p < 0.05; double asterisk, p < 0.01; triple asterisk, p < 0.001; no symbol, p > 0.05. Experiments are all *n* of at least 3. For lipidome analysis only data where differences between control and inulin-treated samples exceeded the 95% confidence interval for three replicates were included in subsequent visualizations or analyses

## Results

### Lipid body accumulation in insulin-treated mast cells and basophils

**Fig 1:**
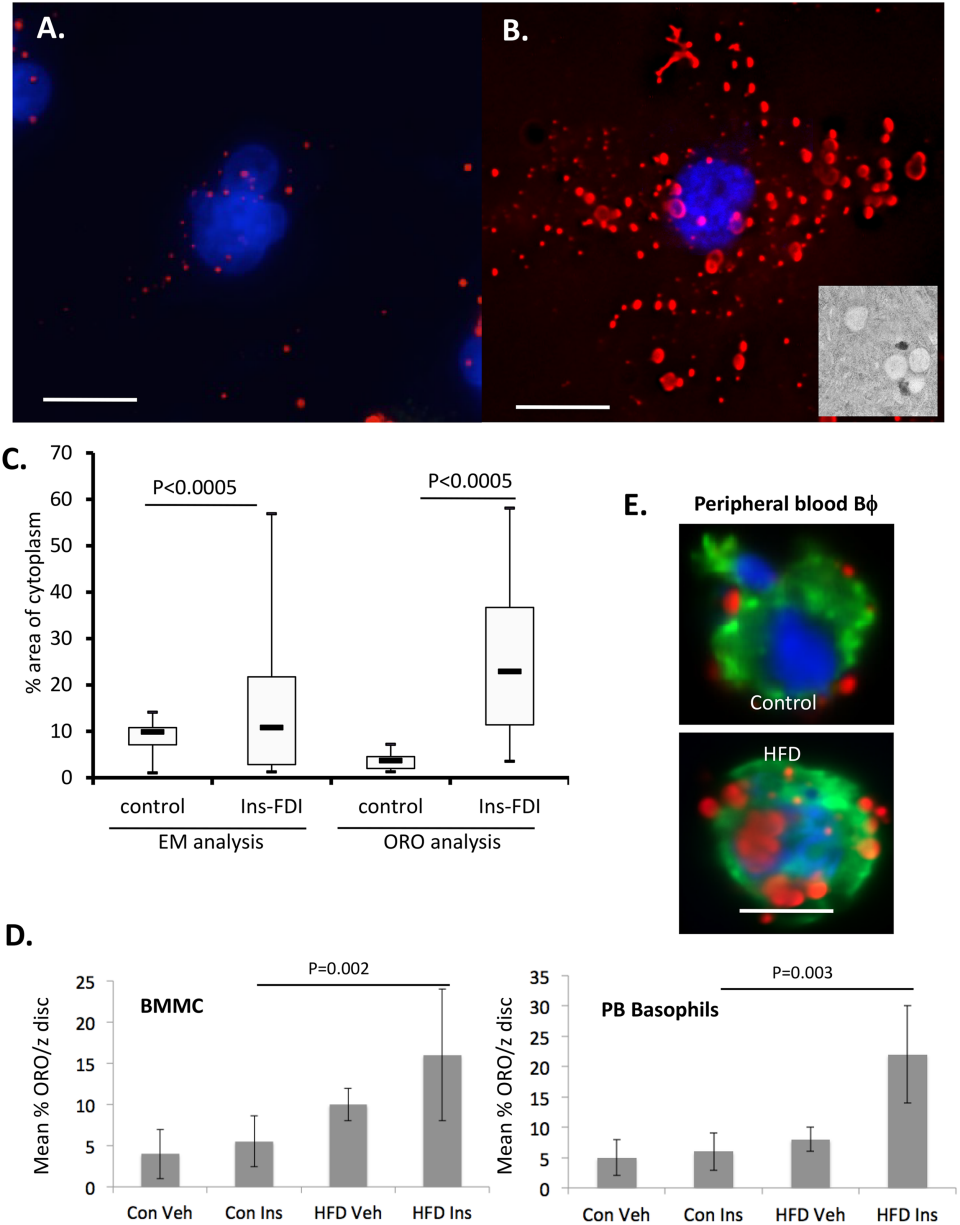
Lipid body induction *in vitro* and *in vivo* by insulin. **A, B.** Fluorescent visualization of lipid bodies using Oil Red O (ORO, 0.35% w/v) counterstained with DAPI in PFA-fixed RBL2H3 cells either untreated (A) or treated with 0.01 mg/ml insulin for 6d (B). Deconvolved z stacks were projected as an extended depth of focus (EDF) image using Nikon NIS Elements. Scale bars are 5 microns. **B.** *inset*. 120,000x electron micrograph image of LB structures from 6d insulin-treated cell. **C.** Area measurement of insulin-induced cytosolic structures were assessed by analyses of EM micrographs (NIH Image J, n = 12) or binary threshold analysis of 25 whole cell ROI for ORO. The whole cell area was determined using a WGA membrane stain and % area of cytoplasm was calculated. The average of the calculations (n = 12 cells for EM analysis and n = 25 cells for fluorescence analysis) are plotted. **D.** ORO assessment of LB numbers in BMMC from normal and HFD-maintained mice. BMMC were prepared as described and maintained for 6d in 200 ng/ml insulin. Z discs (n = 25 z discs from 6 cells) were assessed for ORO stained area and data are expressed as mean % of the z disc (ROI determined by WGA staining for cell boundaries) that was ORO positive. **E.** Murine peripheral blood basophils were isolated using MACS, fixed and stained for LB content as described. Counterstains are DAPI and WGA. 3D rendered images (upper panels) were used to calculate cell volume and the % of cytoplasm (lower panel) occupied by ORO-positive structures. Con, control; Veh, Vehicle; Ins, Insulin. Veh samples are n of only 1, and are shown for comparison purposes only.

We have previously shown that chronic exposure to insulin *in vitro* causes ectopic lipid accumulation in the RBL2H3, a basophilic model that recapitulates many features of mucosal mast cells, and in primary bone marrow derived mast cells (BMMC) [22]. The lipid body (LB) population in these cells is Oil Red O positive and can be visualized by EM (Fig 1A and 1B). Quantitative analysis of the cytosolic abundance of *in vitro* insulin-induced LB visualized either fluorescently or by EM is shown in Fig 1C. We asked whether hyperinsulinemia *in vivo* is sufficient to modulate LB numbers in primary mast cells or the related basophil cell type. Bone marrow derived mast cells (BMMC) from HFD mice treated *ex vivo* for 6d with sub-optimal concentrations of insulin showed LB accumulation relative to controls, as did peripheral blood basophils isolated from HFD fed mice (Fig 1D and 1E). These data confirm that LB numbers are dynamic in mast cells and respond to chronic insulin elevation both *in vitro* and *in vivo*.

### Hyperinsulinemia and high fat diet are associated with altered pro-inflammatory phenotype in primary mast cells

**Fig 2:**
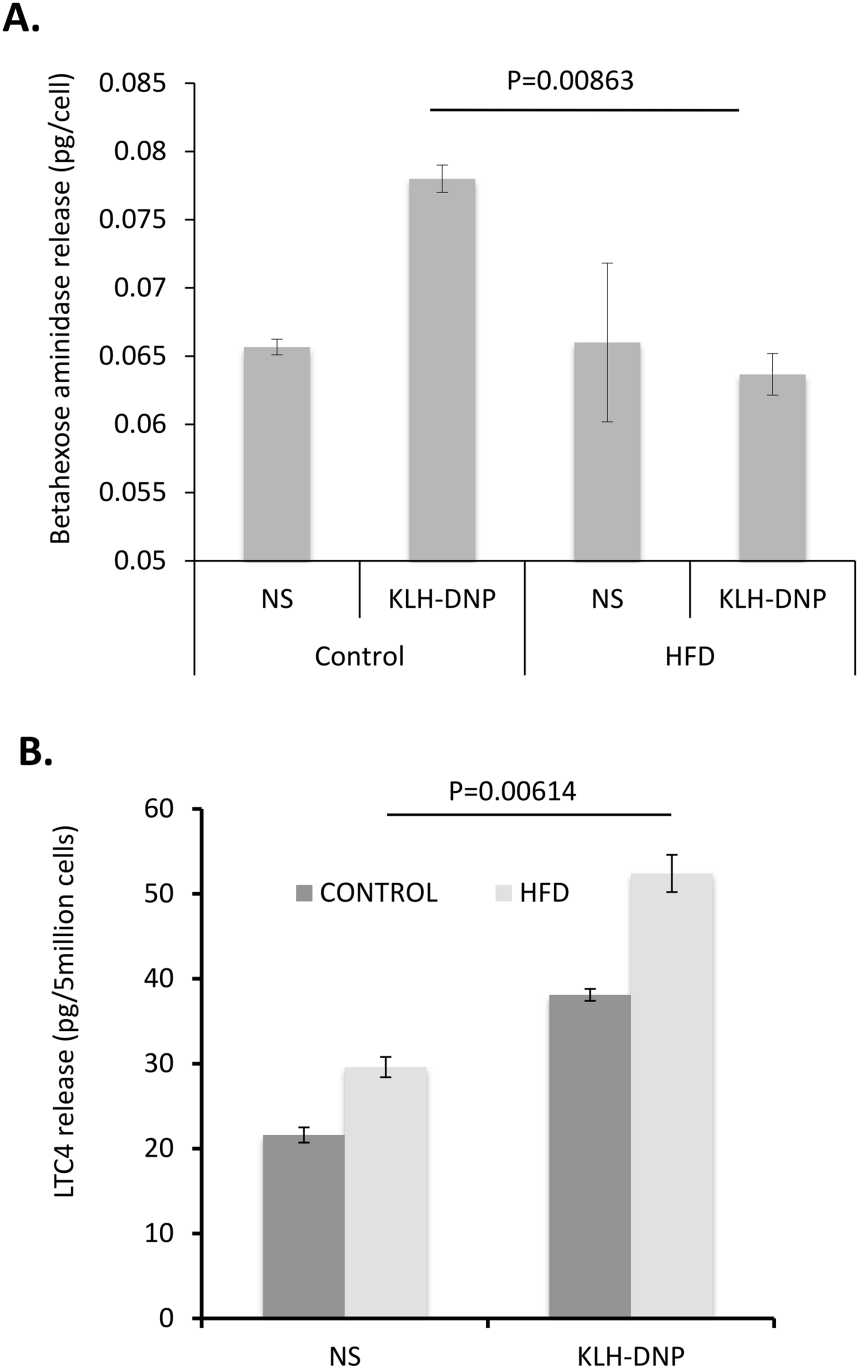
Pro-inflammatory phenotype in mast cells exposed to hyper-insulinemic conditions *in vivo*. **A, B**. Suppressed beta-hexosaminidase release **(A)** and elevated LTC4 **(B)** release in BMMC from C57/BL6 mice maintained on normal or high fat diets (HFD). BMMC were generated from femurs and cultured in IL-3 and sub-optimal (0.5 ng/ml) insulin for 6 d after confirmation of Fc-RIalpha chain staining (not shown). FcεRI stimulation achieved by cross-linking of IgE-bound FcεRI with 200ng/ml KLH-DNP.

In a previous study we have shown that chronic insulin treatment of a model mast cell line or BMMC causes a suppression in secretory granule release and an elevation in LTC4 release, associated with the appearance of a large number of cytosolic LB [22]. Our prior study did not show that this observation was relevant *in vivo* (for example under the conditions of chronic hyperinsulinemia that characterize HFD-induced obesity), and did not provide any direct evidence that the contents of the induced LB were consistent with an enhancement in precursor pools for the production of mediators such as LTC4. To address the first of these questions we asked whether HFD-associated hyperinsulinemia induced elevated LTC4 release. In Fig 2 we compared control and HFD animals (in which elevated plasma insulin was confirmed e.g. 10.1 ng/ml in HFD versus <3ng/ml in controls) for the degranulation and LTC4 release responses of *ex vivo* cultured BMMC. These cells were isolated from either control or HFD animals, differentiated *ex vivo* using IL-3. During the 6 week *ex vivo* differentiation period both control and HFD BMMC were maintained in a sub-optimal concentration of insulin that by itself did not induce LB accumulation (5ng/ml). Relative to cells from control animals, mast cells from HFD animals displayed diminished secretory granule degranulation (Fig 2A) and elevated LTC4 release (Fig 2B) in response to a given dose of antigen. Thus the experiment in Fig 2 shows that HFD itself is sufficient to cause a phenotypic shift in BMMC such that they release elevated LTC4 and exhibit suppressed degranulation responses to antigenic cross-linking of FcεRI. We made a meta-analysis of data across *in vitro* and *in vivo* experiments that support the observation that hyperinsulinemia drive this phenotypic shift. These data are summarized as follows: in 35 experiments (29 *in vitro* and 6 *in vivo*) where the difference between control and insulin-treated or HFD conditions exceeded a 95 percent confidence interval (all p<0.05), we noted a mean of 40.7% enhancement in LTC4 release (SD = 28%) and a mean of a 72.5% decrease in secretory granule release (SD = 19.6%). These data suggest that there is a connection between LB accumulation and the increased intensity of LTC4 release following antigenic stimulation of mast cells. We therefore examined whether a full-scale analysis of the lipid content of these LB could provide any support for this role.

### Lipidome of LB induced by insulin in RBL2H3

**Fig 3:**
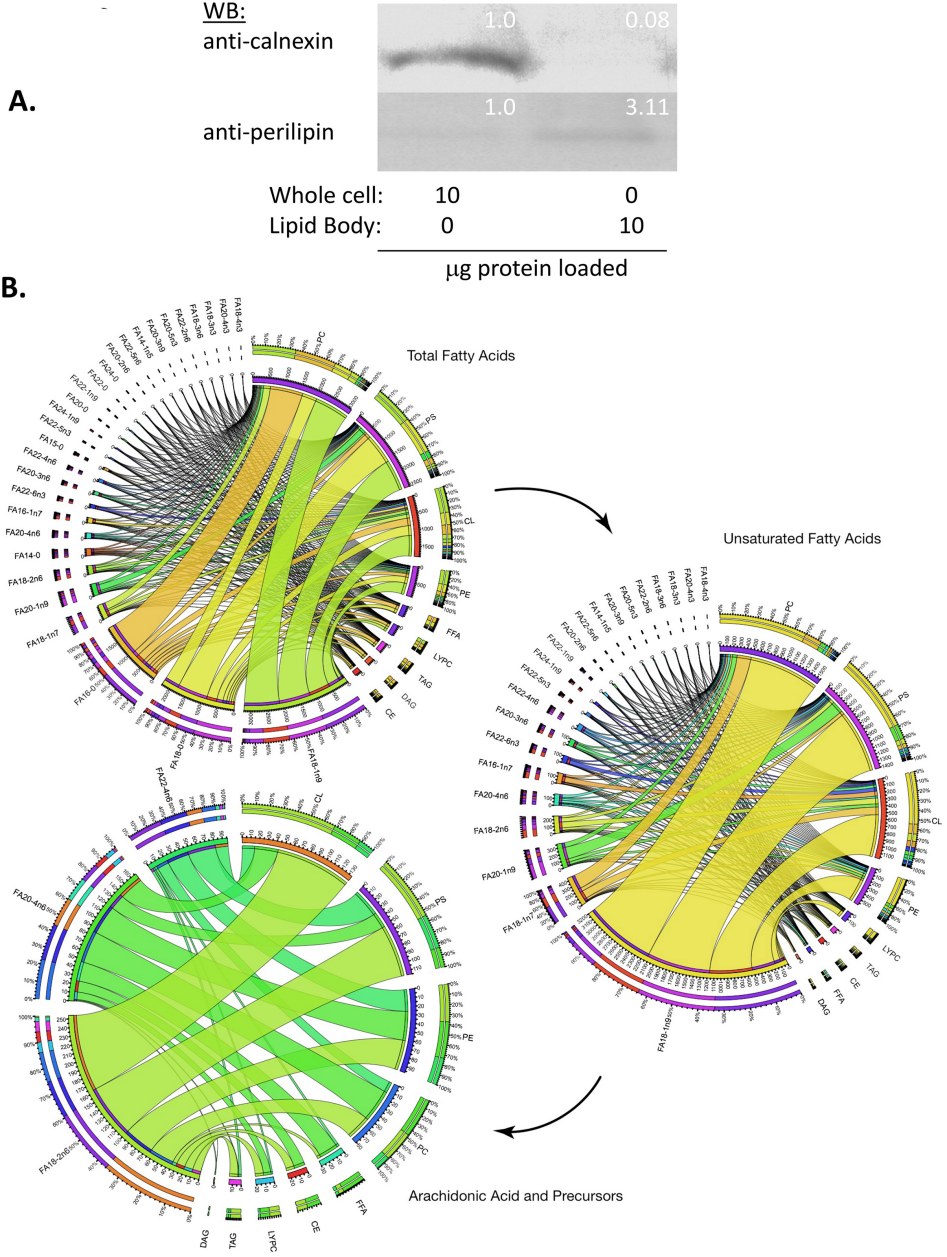
Lipidome of lipid bodies isolated from insulin-exposed RBL2H3. RBL2H3 were grown for 6d with insulin-FDI and LB were isolated by ultracentrifugation. **A. Confirmation of LB enrichment in ultracentrifugation fractions**. Fractions were Western blotted for the enriched presence of the LB-associated protein perilipin A and the diminished representation of the ER-resident chaperone calnexin. Each lane resolves 10 μl of whole cell or LB protein determined by BCA assay. Inset numbers represent ratio of expression determined by integrated density of the Western band (Image J). **B. Lipid profile of isolated LB**. Individual major lipid classes were separated by high performance liquid chromatography (HPLC) and fatty acid methyl esters from each class were produced and subsequently analyzed by GC/MS. Abundance of lipid species in LB from insulin-FDI treated mast cells were quantified and organized into ribbon plots using Circos. All lipid species and nmole percentage representations of the observed classes are visualized. The Circos plots draw ribbons from the fatty acids to the different associated classes. Line width is proportional to the recorded percentage. The outer ring is representative of the total nmole percentage of either the fatty acid and/or class. The inner ring is the relative amount of each element in the plot. The values listed on the inner ring are 100x larger than the percentage in order to resolve less common fatty acids. The three different plots reduce the information from the total fatty acids (top) to the unsaturated fatty acids (middle) to arachidonic acid and its precursors (bottom). This Fig resolves which lipid class and at what percentage each arachidonic acid pathway member occurs.

A comprehensive analysis of lipid content in any mast cell or basophil LB has not previously been published. We isolated LB from LB-rich RBL2H3 using a published density gradient ultracentrifugation method [13]. Isolation of an enriched LB population was confirmed by Oil Red staining (not shown), by relative enrichment in the LB-associated protein perilipin and the concomitant loss of the ER marker Calnexin from the preparations (Fig 3A). We analyzed 342 distinct lipids (38 fatty acids in 9 major lipid classes) using LC/MS and GCMS. Fig 3B shows Circos plot visualizations of lipid content in samples enriched for LB [14, 35]. Overall, 29 fatty acids and 9 major lipid classes were represented. Phosphatidylcholine (PC) (a major component of the unilamellar layer surrounding LB cores), Phosphatidylserine (PS), and Cardiolipin (CL) are highly abundant accounting for roughly 75% of the total lipid class abundance. To assist in visualization, the data are split into 3 plots (Fig 3B), which are sequentially reduced from total fatty acids, to unsaturated fatty acids, to arachidonic acid and its precursors. Fig 3B shows that although the LB are enriched with 16:0 (palmitic) and 18:0 (stearic) saturated fatty acids, the immunologically important 20:4 *cis*-6 (arachidonic) and its precursors 18:2 *cis*-6 (linoleic) and 22:4 *cis*-6 (adrenic) fatty acids are present in four major phosphatidylglycerol lipids: CL, PS, phosphatidylethanolamine (PE), and PC. These membrane-associated lipids are used to recruit precursors in the arachidonic acid cascade and the resulting eicosanoids [36]. In addition to palmitic and stearic acids, 18:1 cis-9 (oleic) monounsaturated fatty acids are also abundant in the LB profile. These three fatty acids comprise at least 60% or higher of all observed fatty acids. Both stearic and palmitic acids are associated with the Toll-like receptor 4 (TLR4) proinflammatory pathway in the context of insulin resistance and obesity [28, 37, 38]. Oleic acid is associated with the activation of the proinflammatory mediator tumor necrosis factor-alpha (TNF-alpha) and the transcription factor nuclear factor kappa-light-chain-enhancer of activated B cells (NF-kappa Β) [39]. S1 Fig breaks out the total fatty acid data into its unsaturated and saturated members and into nine different lipid classes. CL accounts for approximately 18% of the total lipid classes, which may reflect mitochondrial contamination as this lipid is identified with the inner catalytic mitochondrial membrane. However, CL typically accounts for ~20% of those membranes, which would seem insufficient to account for 18% of the total lipid measured in the LB [30, 40]. Interestingly, classes associated with energy storage, namely diacylglycerol (DAG) and triacylglycerol (TAG) were found in low abundance in the LB but are major components of classical lipid droplets found in adipocytes.

### Whole cell lipidome remodeling in insulin-exposed RBL2H3

**Fig 4:**
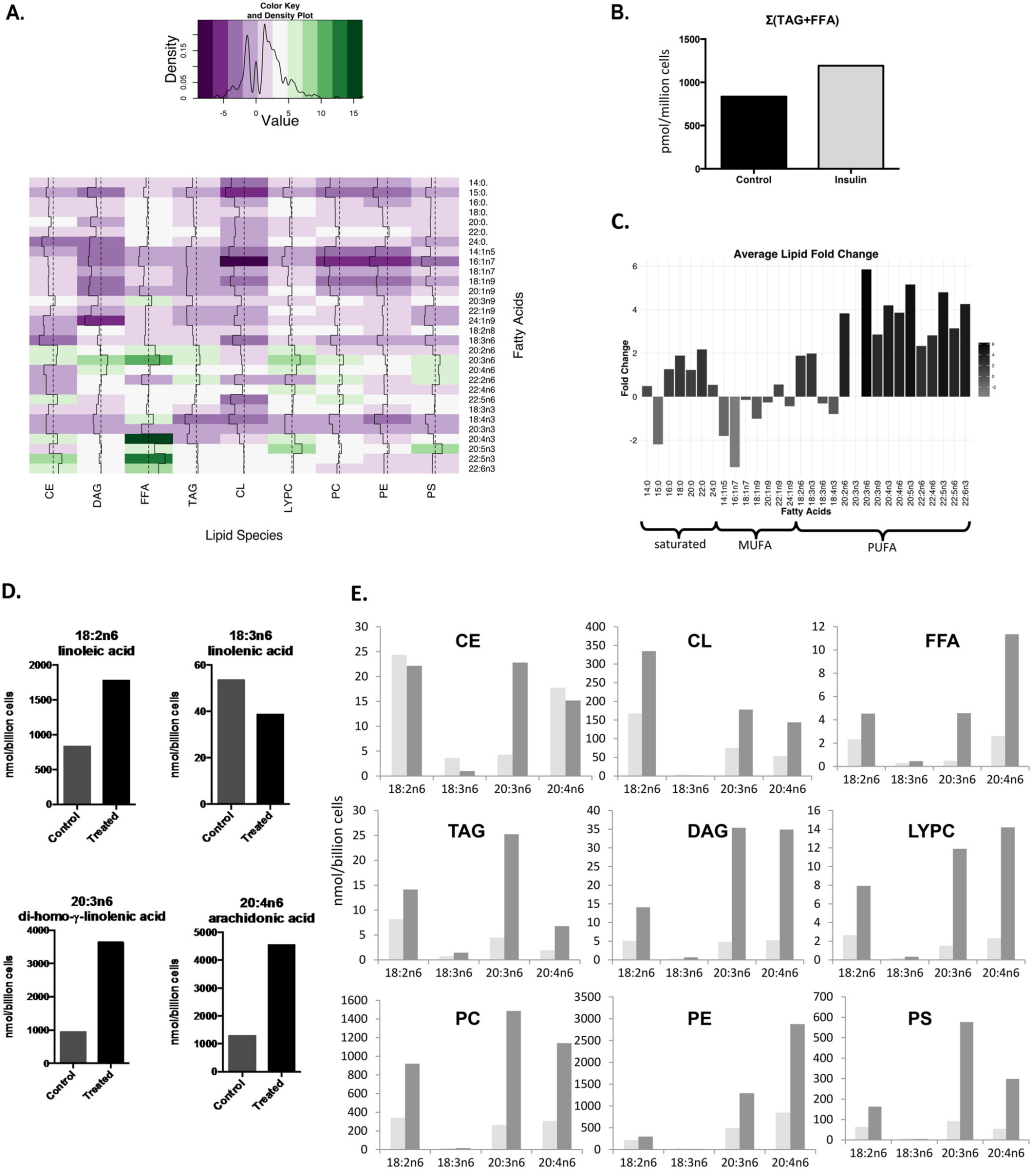
Whole cell lipidome of control and LB-rich RBL2H3. RBL2H3 were grown for 6d with insulin-FDI. Individual major lipid species were separated by high performance liquid chromatography (HPLC) and fatty acid methyl esters (FAME) from each class were produced and subsequently analyzed by GC/MS. **A.** The total fold change in nanomolar amounts of each fatty acid within each class of lipids are presented in a heatmap using RColorBrewer and gplots in R. Negative fold change moves towards purple whereas positive fold change moves toward green. Trace lines are used to reinforce change within the groups and are relative to a dashed median line. The density of change is tracked within the scale bar to the left. **B.** Change in absolute TAG and FFA levels summed between untreated and insulin-treated cell samples. **C**. The average fold change of fatty acids between the two conditions is bar plotted and organized by the degree of saturation (saturated, mono-unsaturated (MUFA), and poly-unsaturated (PUFA)) of the fatty acids. The legend on the right side indicates the scale in fold change from -3.5 to 6. **D.** Individual FA in the AA biosynthetic pathway were quantified according to their respective major lipid class. Fold change was calculated based on response to insulin-FDI in treated compared to control mast cells (18:2n6, linoleic acid; 18:3n6, linolenic acid; 20:3n6, di-homo-gamma-linolenic acid; 20:4n6, arachidonic acid). **E.** Experiment as in **D**, with quantification of individual FA directly involved in the AA biosynthesis pathway quantified by lipid class in terms of absolute concentration (nmol of lipid per billion cells). *Cholesterol Ester (CE)*, *Cardiolipin (CL)*, *Triacylglycerol (TAG)*, *Diacylglycerol (DAG)*, *Free Fatty Acid (FFA)*, *Phosphatidylserine (PS)*, *Phosphatidylcholine (PC)*, *Phosphatidylethanolamide (PE)*, *Lysophoshatidylcholine (LYPC)*.

Density gradient centrifugation yields a highly enriched population of LB, and the very high abundance of LB in insulin-exposed cells minimizes the quantitative contribution of contamination (e.g., with LB-associated mitochondria or similarly sedimenting microsomal vesicles). In untreated cells, the low abundance of LB made this contamination more significant and enrichment difficult to validate. We therefore examined whole cell lipidomes to compare untreated and treated cells, reasoning that the abundance of insulin-induced LB would translate to an impact on the overall total lipid signature. Whole cell lipid extracts from insulin-exposed RBL2H3 show alterations in major lipid classes and associated fatty acid composition (Fig 4A). Of the 9 major lipid classes examined, we find free fatty acids (FFA) and lysophosphatidylcholine (LYPC) increase more than two fold with marginal increases in 4 of the other major lipid classes (CE, TAG, PS, FC). A survey of individual FFA show the largest increase in eicosatetraenoic acid (20:4n3, 16.3-fold) and the largest decrease in palmitoleic acid (16:1n7, 8.9-fold change). Several omega-3 fatty acids are upregulated over 10-fold (e.g., 20:4n3 and 22:5n3). Di-homo-gamma-linolenic acid (20:3n6), the immediate precursor molecule for arachidonic acid (AA), increased across all major lipid classes at 5.85 fold, with eicosapentaenoic acid (20:5n3) at 5.15 fold. In terms of absolute abundance of lipid species, we find the most marked alterations in 22:6n3 and 20:3n6 (a precursor to AA), and AA itself. Overall levels of TAG and FFA, conventionally regarded as major components of adipocyte lipid droplets, are not strongly upregulated (Fig 4B).

We detected alterations in the degree of unsaturation in individual fatty acids (Fig 4C). Insulin-FDI treatment resulted in upregulation of omega-3 and omega-6 polyunsaturated fatty acids (PUFA), in particular the 18–20 carbon omega-6 PUFAs, which include precursors for AA synthesis. Fig 4C includes intermediates involved in the biosynthesis of AA. With the exception of 18:3n6 (a short-lived metabolite in this biosynthetic pathway), our data show an upregulation in each FA in all 9 major lipid classes in the AA pathway. Absolute levels increased in 3 of 4 lipids of the AA biosynthetic pathway (Fig 4D). AA showed > 3-fold increase over control levels in 7 of the 9 major lipid classes (Fig 4E), and we were able to validate this finding by pan-AA ELISA (data not shown).

**Fig 5:**
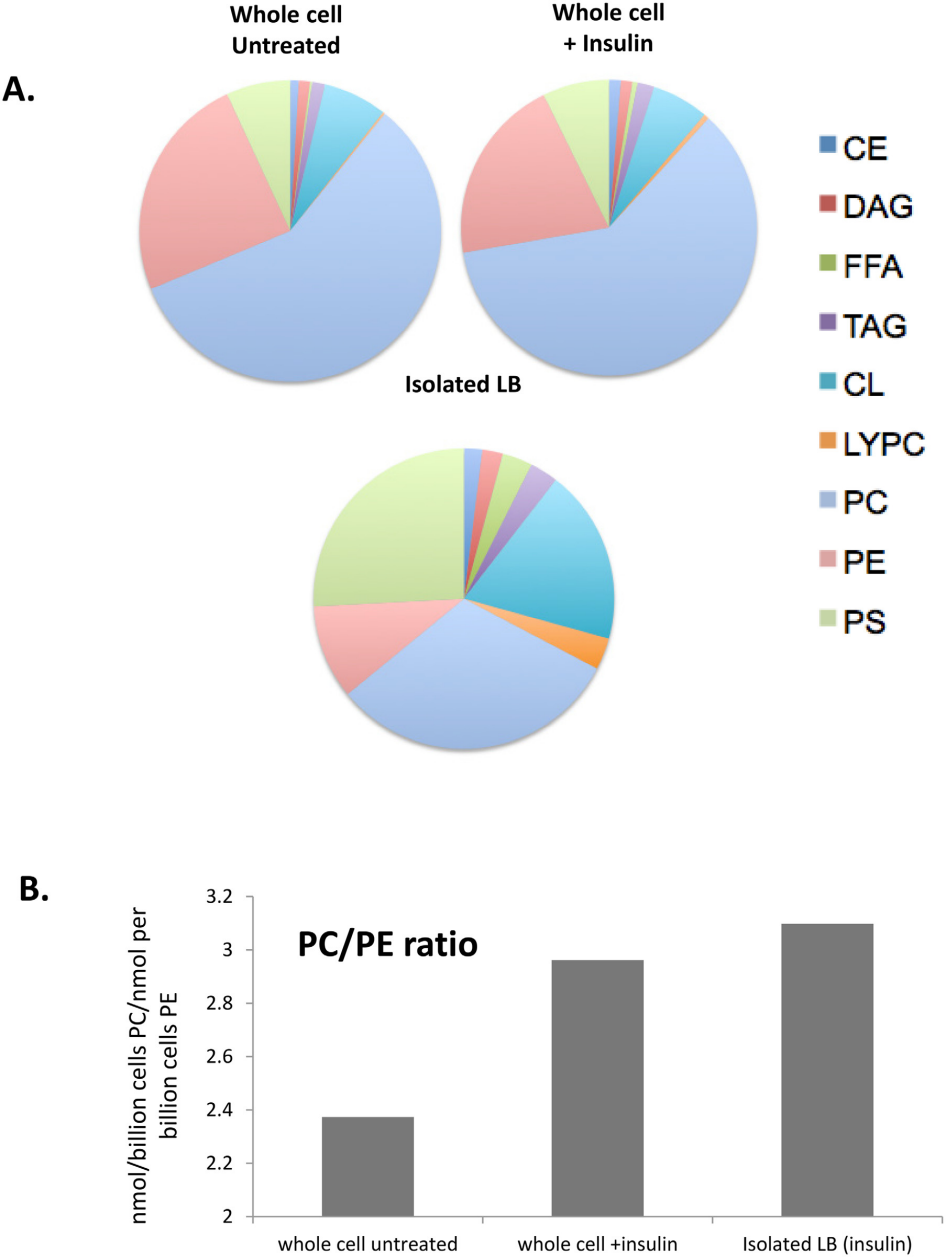
Representation of lipid species in isolated LB and whole cell lipidomes. **A.** Comparison of the percentage abundance of the indicated lipid classes in the whole cell extracts from untreated and insulin (6d) treated cells, and isolated LB from 6d insulin-treated cells. **B.** PC/PE ratio calculated for whole cell extracts from untreated and insulin (6d) treated cells, and isolated LB from 6d insulin-treated cells. *Cholesterol Ester (CE)*, *Cardiolipin (CL)*, *Triacylglycerol (TAG)*, *Diacylglycerol (DAG)*, *Free Fatty Acid (FFA)*, *Phosphatidylserine (PS)*, *Phosphatidylcholine (PC)*, *Phosphatidylethanolamide (PE)*, *Lysophoshatidylcholine (LYPC)*.

Fig 5 draws together data from the whole cell and isolated LB lipidomes. We assessed (Fig 5A) the relative over- or under-representation of lipid species in LB from LB-rich cells. Organizing these data by lipid class, we note overall similarity between control and insulin-treated whole cells. The LB signature is markedly different, with over-representation of FFA, CL and PS relative to whole cells. Notably DAG, TAG and CE are not markedly over-represented in isolated LB. PE is under-represented in LB relative to whole cells. We calculated the PC/PE ratio for whole cells and isolated LB (Fig 5B). PC expansion is consistent with the elevated need for this lipid to coat the surface of a large population of new LB was noted in insulin treated cells. Moreover, PC is highly represented in the purified LB, again reflecting its role in formation of the unilamellar coat enclosing the LB [25, 29].

### Insulin-induced LB accumulation is accompanied by ER reprogramming in mast cells

**Fig 6:**
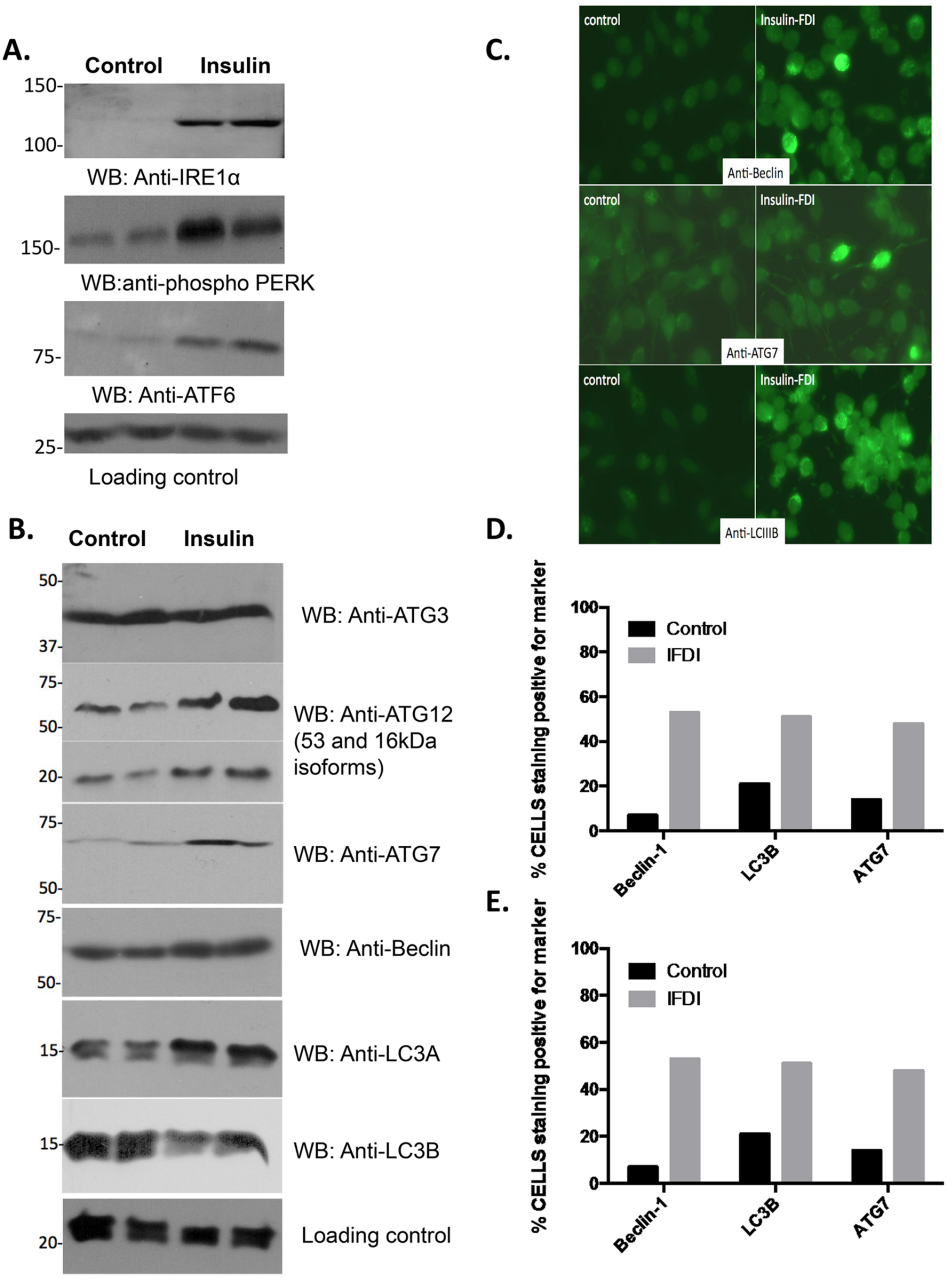
ER reprogramming, ER stress and autophagy in insulin-treated RBL2H3. **A, B. Altered expression of markers of ER stress, the UPR and autophagy.** RBL2H3 were treated with insulin-FDI for 6d and protein lysates were prepared. Western blot analysis (antibody concentrations indicated in micrograms/ml)was performed using UPR markers anti-IRE1 alpha (0.5), anti-phospho PERK-Thr980 (0.1), anti-ATF6 (2.5) and loading control anti-GRB2 (0.05)(A) and autophagy markers (B) anti-ATG3 (0.5), anti-ATG12 (0.5), anti-ATG7 (0.5), anti-Beclin (0.5), anti-LC3A (0.1), and anti-LC3B (0.5) with anti-Grb2 as a loading control. **C-E.** Immunofluorescent identification and quantification of autophagy positive mast cells. Three markers of autophagy (Beclin-1, LC3B and ATG7) were used to quantify the percent of cells staining positively for autophagy (**C**). **D, E**. Quantification of autophagy marker immunostaining. Counting was performed in a sample-blinded fashion and expressed as % of 200 counted cells (**D**) and mean of the number of immunodecorated structures per cell (**E**).

LB accumulation in insulin-exposed mast cells would be predicted to affect the ER due to the intimate relationship between the ER and LB biogenesis [25]. Steatosis in the liver causes ER ‘reprogramming’, characterized by *(i)* ER stress, *(ii)* the Unfolded Protein Response (UPR), *(iii)* ER distension, and *(iv)* a shift from predominantly protein synthesis to lipid biogenesis [41]. We assessed these responses in cells exposed chronically to insulin. Fig 6A shows that various markers of the UPR (IRE1 alpha, phospho-PERK and ATF6) are upregulated in cells exposed chronically to insulin. Ideally, the UPR protects cells against ER stress by enhancing the capacity of the secretory apparatus and by reducing the ER load [42]. However, dramatic dysregulation of the ER can lead to autophagy where the stressed ER is engulfed by autophagosomes in an attempt to regain homeostasis. Autophagy-related proteins (ATG3, ATG12, ATG7, Beclin, and LC3A) were upregulated in insulin-treated RBL2H3 (Fig 6B) [43]. This suggests that both the UPR and autophagy responses are induced by the degree of ER stress activated in insulin-treated RBL2H3 cells, and we validated this observation by immunocytochemistry of autophagy-related proteins (Fig 6C, 6D and 6E).

**Fig 7:**
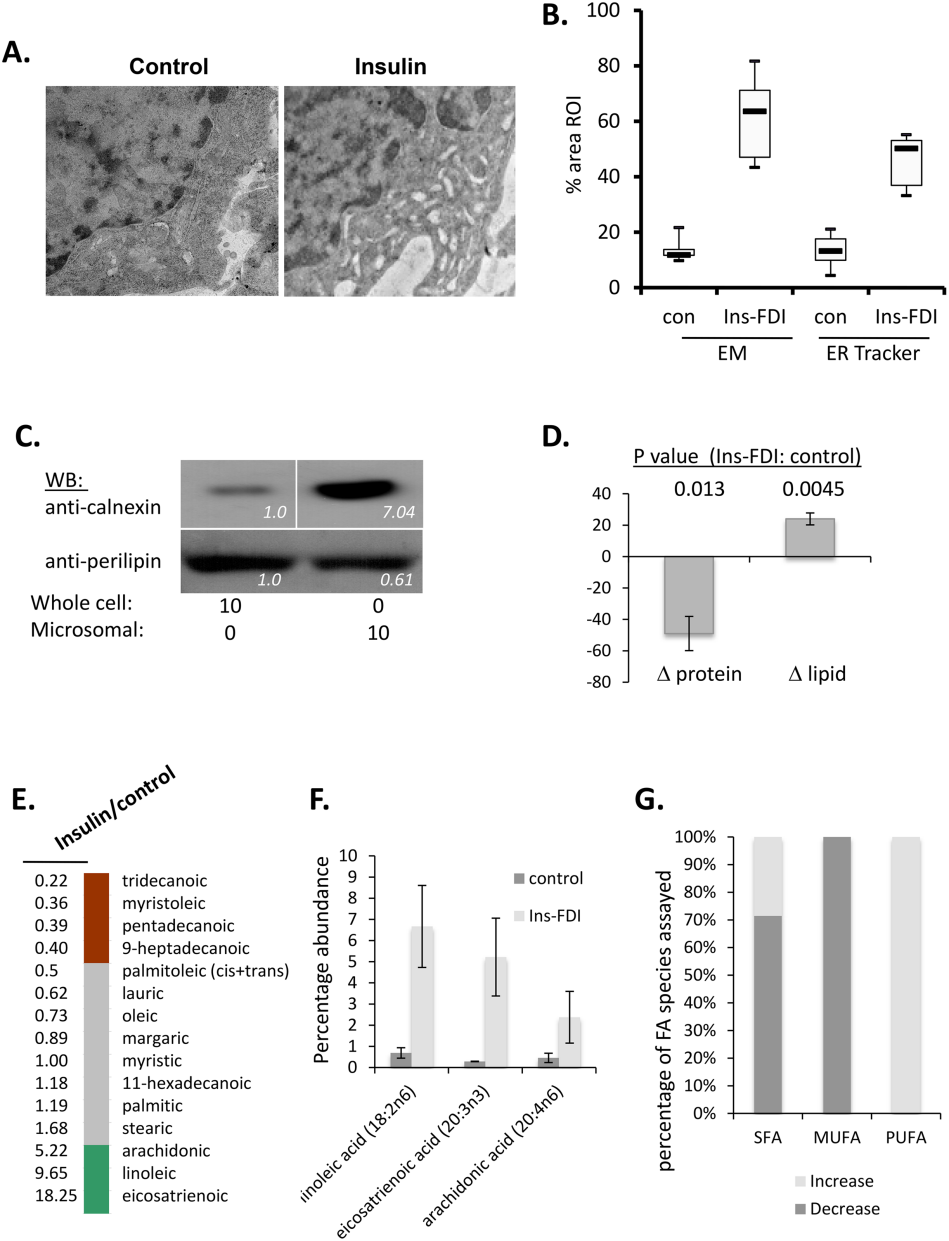
ER distension and lipid biogenesis in insulin-exposed RBL2H3. **A. Electron micrographs of control and insulin-FDI exposed RBL2H3 showing normal (left) and distended (right) ER.** Images are digitally zoomed from 5000x original plates. **B. Area analysis of ER by electron and fluorescence microscopy**. Cytoplasmic (n of 20 per condition) ROI were drawn on either micrographs (Image J) or confocal images of cells stained with ER-Tracker dye (NIS Elements). Data are expressed as percentage area of ROI occupied by ER. **C. Confirmation of ER enrichment in purified ER/microsomal fractions.** ER/microsomal fractions were prepared by ultracentrifugation as described in Methods and Western blotted for enrichment in the ER-resident chaperone Calnexin. Relative band intensities are shown on each panel (Image J). **D. Comparison of protein and lipid levels in ER/microsomal fractions prepared from control and Insulin-FDI treated RBL2H3**. Total protein was assessed by BCA analysis, and total lipid content was assessed by ORO absorbance assay after Bligh-Dyer extraction. **E. Characterization of ER Fatty acids**. RBL2H3 were grown for 6d with insulin-FDI as described. Isolated ER was analyzed by GC/MS and variations in ER lipids versus control levels were organized by fold change (values along y-axis) and abundance. *Green*, > 2 fold increase in treated over controls; *gray*, no change; *red*, decrease to < 50% of control levels. **F. Relative abundance of eicosatrienoic, arachidonic and linoleic acid in ER from control and Insulin-FDI treated cells**. **G. Summary of alterations in saturated (SFA), mono-unsaturated (MUFA) and poly-unsaturated (PUFA) fatty acids in ER/microsomal fractions from control and insulin FDI-treated cells**.

ER lamellae are distended in insulin-FDI treated cells, with close agreement between both EM and fluorescence-based assessments of ER-area (Fig 7A and 7B). The Hotamisligil laboratory suggested that such ER remodelling is associated with ER reprogramming in the obese liver, where steatosis is associated with lipid accumulation in the ER and diminished ER protein synthesis [29]. We therefore generated enriched ER/microsomal fractions by ultracentrifugation (Fig 7C) and assessed their overall lipid and protein content in control and LB-rich mast cells (Fig 7D). The ER in LB-rich mast cells is in a similar state to that of the obese liver, with a net increase in lipid, and decrease in protein content [29]. LB biogenesis is initiated by the accumulation of lipid ‘lenses’ between the ER membrane lamellae [44]. In cells where LB are major storage sites for ER-derived TAG, it would be predicted that the stressed ER in LB-rich cells would have a lipid content closely related to that of the LB themselves. We analyzed the lipid content of ER/microsomal fractions purified from control and LB-rich mast cells by density gradient ultracentrifugation. We observe the most abundant fatty acids in our ER isolation to be palmitic acid (16:0), stearic acid (18:0) and oleic acid (18:1n9), which are the same 3 fatty acids with the highest abundance analyzed from our LB fractions. We noted redistribution in the relative abundance of individual fatty acids in the samples derived from LB-rich cells (Fig 7E and 7F). We again noted upregulation in AA precursors and AA itself compared to untreated cell ER fractions (Fig 7F). We also note changes in all degrees of saturation in ER lipid content in Insulin-treated cells including saturated, mono- and poly-unsaturated fatty acids (Fig 7G).

## Discussion

Our data suggest that high fat diet (HFD) exposure and hyperinsulinemia are associated with LB accumulation, suppressed degranulation and enhanced leukotriene release in both BMMC and peripheral blood basophils. These data support and extend previous *in vitro* findings. Chronic elevated insulin and nutrient overload can thus be added to the short list of stimuli that have been shown to regulate LB numbers in immunocytes, alongside bacterial and parasite components, cytokines, extracellular FA, and platelet activating factor (PAF) [6]. The only hormones previously shown to regulate LB numbers in an immune system cell (foamy macrophages) are resistin and leptin [6, 13]. It now remains to be seen whether immunological inputs also regulate LB numbers in mast cells, in addition to the endocrine input of insulin. Insulin has previously been suggested to be a survival factor for BMMC, which may be an important factor in HFD/hyperinsulinemic animals. However, in our studies neither RBL2H3 [22] or primary BMMC showed positive effects of insulin on proliferation.

Both *de novo* lipid biogenesis and the import of excess dietary lipid (stored cytoplasmically as TAG/CE) from the bloodstream have been implicated in steatosis in adipocytes and hepatocytes [29]. Our lipidomic analysis suggests that the lipidome of LB-rich mast cells may reflect a more complex picture, where significant expansion occurs in lipid species that are precursors to the biosynthesis of leukotrienes and prostaglandins. This analysis of isolated LB provides the first fingerprint of these organelles in terms of lipid content, albeit in an expanded LB population that may, or may not, directly reflect the content of the LB in untreated naïve mast cells, or those in tissue. Notable features of the LB lipidome here are the representation of AA and its biosynthetic precursors, and very high levels of PC. The former is consistent with previous EM and biochemical studies, showing that mast cell LB are sites for AA metabolism and LT synthesis [11, 12]. The latter is consistent with the role of PC as the primary component of the unilamellar coat of the LB surface [45]. Fu *et al* also identified a large shift in the PC:PE ratio in hepatocytes that were accumulating lipid droplets [29], and reasoned similarly. Like the induction of ER stress and UPR, this is another commonality between the obese liver and the insulin-exposed immunocytes studied here, where we noted transcriptional upregulation of PC synthesizing enzymes (*pcyt* and *pemt*, not shown) and the presence of ~40% more PC than in control cells (note that the surface area of a spherical lipid body of 1 micron diameter would approximate 3x10^-12^m^2^, perhaps necessitating synthesis of some 3 million ~1.5 nm^2^ PC headgroups). It should be noted however, that the ultracentrifugation method of LB purification may be subject to contamination by associated organelles or those of similar sedimentation properties. For example, the high levels of cardiolipin (CL) observed in purified LB could derive from mitochondria that have been observed to closely associate with some LB in other cell types. However, the high levels of CL that we observe, and the interesting lipid profile within this class (S1 Fig) suggest that CL levels may not simply be reflective of this contamination. Further validation of the CL presence in LB, and study of its role, is clearly required. We note that it is also difficult to discriminate between lipids from LB and from the ER that is either closely associated with them [6, 13], or that is residual from the LB biogenesis process. We have therefore embarked on microaspiration and nanospray LC/MS lipid analysis of LB as a method to allow us to study an even more purified population.

Expanded LB pools in adipocytes and hepatocytes from obese animals are also associated with remodelling of the cellular lipidome and the concomitant induction of ER stress. ER remodelling and reprogramming (from protein synthesis to lipogenesis) that has been observed in the obese steatotic liver is also, interestingly, paralleled in the immune cells studied here [29, 45]. In parallel with studies in the obese liver, we found an upregulation of the UPR pathway and autophagy genes in the LB-rich mast cell. In this case, autophagy induction is likely to be an outcome of the lipid saturation of the ER, with autophagosome activity attempting to regain homeostasis. While acute insulin can protect against autophagy in some cell types, the relationship in our study is likely to be indirect, with autophagy resulting from the effects of chronic insulin elevation on lipid accumulation.

Isolated adipocytes and macrophages from obese tissue show large increases in PUFA and TAG levels [19, 46, 47]. Under the hyperinsulinemic conditions mimicked in our experiments, RBL2H3 show dramatic increases in PUFA but marginal changes overall in TAG. The increase in PUFA that we observe, if translated to mast cells found *in vivo*, could provide a new source for the PUFA that are dramatically elevated in adipose tissue. Obese adipose is enriched in mast cells, enriched in PUFA and is inflamed. A population of resident mast cells in obese adipose that is producing PUFA and also elevated levels of some mast cell-derived pro-inflammatory mediators such as eicosanoids, could contribute to this phenotype. Elevated LB numbers in adipose or other mast cells in response to insulin or obesogenic stimuli is, as yet, unstudied, although Theoharides has alluded to elevated sudan black-positive (a LB dye) structures in mucosal mast cells from obese mice [48].

Ectopic lipid deposition (ELD) occurs outside the classical adipose depots in obesity (e.g., liver, pericardium and skeletal muscle [49]). Our data suggest that cells of the immune system may be sites of ELD in response to nutrient overload, but that the specific character of the accumulated LB is distinct from those in adipocytes (primarily TAG/CE ported from blood) and is more closely related to the presumed physiological role of the endogenous LB in the immune context. Interestingly, while their functional roles are divergent, this suggests commonalities in the basic LB biogenesis mechanism between adipocytes and mast cells, in that insulin can drive the process in both cell types.

Our data suggest that mast cells with an expanded LB population are fundamentally reprogrammed compared to normal cells. LB biogenesis is accompanied by marked changes in their functional responses to antigen. Primary mast cells with expanded LB pools display elevated release of LTC4 after FcεRI cross-linking but their secretory degranulation responses (histamine release) are markedly suppressed. Our staining data (not shown) suggest that the latter relates to a suppression of secretory granule biogenesis rather than a defect in granule cargo or loading. This may relate to the autophagy phenotype—since lysosomes are part of the biogenesis pathway of both secretory granules and autophagosomes, one of these organelles may form at the expense of another.

Exposure to acute innate stimuli has been suggested to cause leukotriene release that is independent of degranulation in mast cells [38, 50]. Our data suggest that mast cells can be ‘pre-programmed’ with a bias towards one of these functional responses, in this case by exposure to high insulin levels. There may be a relationship between these observations and the long-established functional heterogeneity of mast cell sub-populations *in vivo*. Moreover, this study suggests that outcomes of inflammatory responses driven by mast cells would differ in hyperinsulinemic and high nutrient conditions compared to metabolically normal contexts, or in conditions of insulin paucity.

## Supporting Information

S1 Fig Lipid profile of isolated LB by lipid class. Individual major lipid classes were separated by high performance liquid chromatography (HPLC) and fatty acid methyl esters from each class were produced and subsequently analyzed by GC/MS. Abundance of lipid species in LB from IFDI-treated mast cells were quantified and organized into ribbon plots using Circos. All lipid species and nmole percentage representations of the observed classes are visualized. The Circos plots draw ribbons from the fatty acids to the different associated classes. Line width is proportional to the recorded percentage. The outer ring is representative of the total nmole percentage of either the fatty acid and/or class. The inner ring is the relative amount of each element in the plot. The values listed on the inner ring are 100x larger than the percentage in order to resolve less common fatty acids. The top row shows the complete division of fatty acids and lipid group membership. This row is further reduced to either the unsaturated fatty acids or the saturated fatty acids. Subsequent rows show a breakout of each lipid class and the fatty acid membership and percentage for that class. They are ordered from left to right and then top to bottom by percentage abundance of the specific lipid class. By order in the Fig: *Phosphatidylcholine (PC)*, *Phosphatidylserine (PS)*, *Cardiolipin (CL)*, *Phosphatidylethanolamide (PE)*, *Lysophoshatidylcholine (LYPC)*, *Triacylglycerol (TAG)*, *Cholesterol Ester (CE)*, *Free Fatty Acid (FFA)*, *Diacylglycerol (DAG)*.
(TIFF)
