# Supplementary figures and images for: Chronic Insulin Exposure Induces ER Stress and Lipid Body Accumulation in Mast Cells at the Expense of Their Secretory Degranulation Response
Source: PLoS One. 2015 Aug 11;10(8):e0130198. doi: 10.1371/journal.pone.0130198 (PMC4532411; doi:10.1371/journal.pone.0130198)

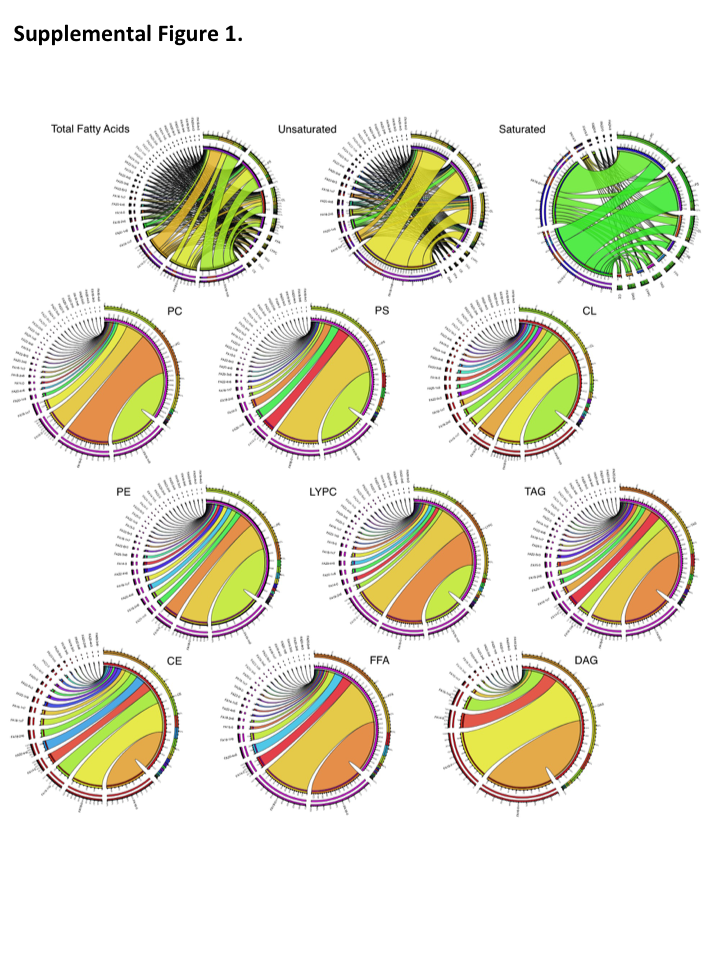

Supplement: S1 Fig — Individual major lipid classes were separated by high performance liquid chromatography (HPLC) and fatty acid methyl esters from each class were produced and subsequently analyzed by GC/MS. Abundance of lipid species in LB from IFDI-treated mast cells were quantified and organized into ribbon plots using Circos. All lipid species and nmole percentage representations of the observed classes are visualized. The Circos plots draw ribbons from the fatty acids to the different associated classes. Line width is proportional to the recorded percentage. The outer ring is representative of the total nmole percentage of either the fatty acid and/or class. The inner ring is the relative amount of each element in the plot. The values listed on the inner ring are 100x larger than the percentage in order to resolve less common fatty acids. The top row shows the complete division of fatty acids and lipid group membership. This row is further reduced to either the unsaturated fatty acids or the saturated fatty acids. Subsequent rows show a breakout of each lipid class and the fatty acid membership and percentage for that class. They are ordered from left to right and then top to bottom by percentage abundance of the specific lipid class. By order in the Fig: Phosphatidylcholine (PC), Phosphatidylserine (PS), Cardiolipin (CL), Phosphatidylethanolamide (PE), Lysophoshatidylcholine (LYPC), Triacylglycerol (TAG), Cholesterol Ester (CE), Free Fatty Acid (FFA), Diacylglycerol (DAG). (TIFF) [file pone.0130198.s001.tiff]
